# A rare pelvic mass in an adult woman: case report of a mature sacrococcygeal teratoma

**DOI:** 10.1093/jscr/rjag158

**Published:** 2026-03-17

**Authors:** Asmae Guennouni, Ihssane Laasri, Soukaina Bahha, Youssef Omor, Rachida Latib, Sanae Amalik

**Affiliations:** Department of Radiology, National Institute of Oncology, University Hospital Center Ibn Sina, Rabat, Morocco; Department of Radiology, National Institute of Oncology, University Hospital Center Ibn Sina, Rabat, Morocco; Department of Radiology, National Institute of Oncology, University Hospital Center Ibn Sina, Rabat, Morocco; Department of Radiology, National Institute of Oncology, University Hospital Center Ibn Sina, Rabat, Morocco; Department of Radiology, National Institute of Oncology, University Hospital Center Ibn Sina, Rabat, Morocco; Department of Radiology, National Institute of Oncology, University Hospital Center Ibn Sina, Rabat, Morocco

**Keywords:** sacrococcygeal teratoma, magnetic resonance imaging (MRI), pelvic cystic lesion, pelvic mass

## Abstract

Sacrococcygeal teratomas (SCTs) are rare germ cell tumors that predominantly occur in neonates and infants, with adult presentations being exceptionally uncommon. These tumors arise from pluripotent embryonic germ cells located in the coccygeal region and may contain a mixture of cystic, solid, fatty, and calcified components. Malignant transformation, although rare, is associated with increased tumor vascularity and aggressive behavior. We present the case of a 30-year-old woman with no prior medical history who presented with urinary incontinence. Initial ultrasound revealed a non-specific cystic pelvic mass, prompting further evaluation with pelvic magnetic resonance imaging (MRI). MRI demonstrated a well-circumscribed presacral mass with typical features of SCT, including cystic components and fatty areas showing signal suppression on fat-saturated sequences, with no evidence of invasion or malignancy. The patient underwent surgical excision, which remains the treatment of choice, and histopathology confirmed a benign mature teratoma. This case highlights the importance of including sacrococcygeal teratoma in the differential diagnosis of adult pelvic masses and reinforces the essential role of MRI in lesion characterization and preoperative planning.

## Introduction

Sacrococcygeal teratomas are uncommon congenital tumors, with adult presentations being particularly rare. They originate from residual pluripotent stem cells in the presacral region, capable of differentiating into various somatic tissue types [1]. The exact etiology remains unclear. Although the majority of teratomas are benign, ~1%–2% may undergo malignant transformation into squamous cell carcinoma, adenocarcinoma, sarcoma, or other malignancies. Sacrococcygeal teratomas typically exhibit slow growth and are often asymptomatic in early stages, leading to delayed diagnosis. In many cases, these tumors are discovered incidentally during physical examination or become clinically evident when mass effect produces compressive symptoms [2].

Advances in magnetic resonance imaging (MRI) and computed tomography (CT) imaging have significantly improved the ability to distinguish benign from malignant presacral tumors based on characteristic imaging features [3].

## Case report

A 30-year-old woman with no significant medical history presented with urinary incontinence evolving over several weeks. She reported no gastrointestinal or neurological symptoms. Clinical examination revealed a conscious, afebrile patient who was hemodynamically and respiratorily stable. Abdominal examination identified a large abdominopelvic mass, firm and slightly mobile on palpation.

**Figure 1:**
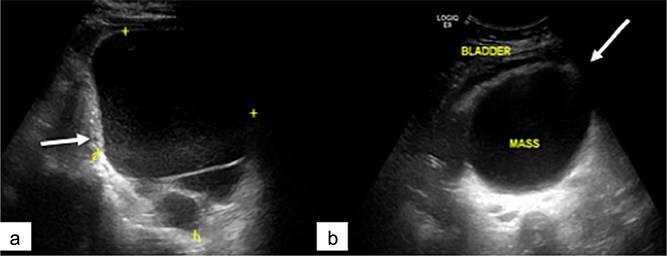
Pelvic ultrasound showing a large multilocular pelvic mass (arrow), displacing the bladder anteriorly.

Abdominopelvic ultrasound revealed a large cystic mass occupying the abdominopelvic region and extending posteriorly, displacing the bladder anteriorly (Fig. 1). The lesion was multilocular and showed no significant vascularization on Doppler imaging.

**Figure 2:**
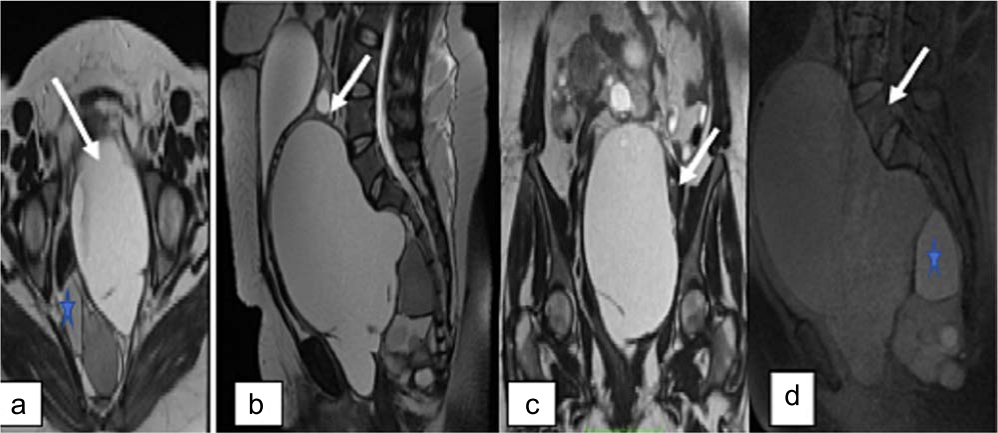
Pelvic MRI. Axial (a), sagittal (b), and coronal (c) T2-weighted images, and sagittal T1-weighted image showing a large multiloculated sacrococcygeal mass containing cystic areas with low T1 signal and high T2 signal (arrow), hemorrhagic components, and fatty components with high T1 and T2 signal (asterisk). No diffusion restriction or contrast enhancement is observed after gadolinium administration.

Pelvic MRI was performed for further characterization. It demonstrated a well-circumscribed, multiloculated presacral mass. The lesion appeared hypointense on T1-weighted images and hyperintense on T2-weighted sequences. It contained cystic areas with high signal intensity on both T1 and T2 sequences, as well as thick septations that showed no enhancement following gadolinium administration. The mass displaced the uterus and bladder anteriorly and was clearly separated from both ovaries, which appeared normal in morphology and signal intensity (Fig. 2).

No signs of local invasion or malignant transformation were identified.

**Figure 3:**
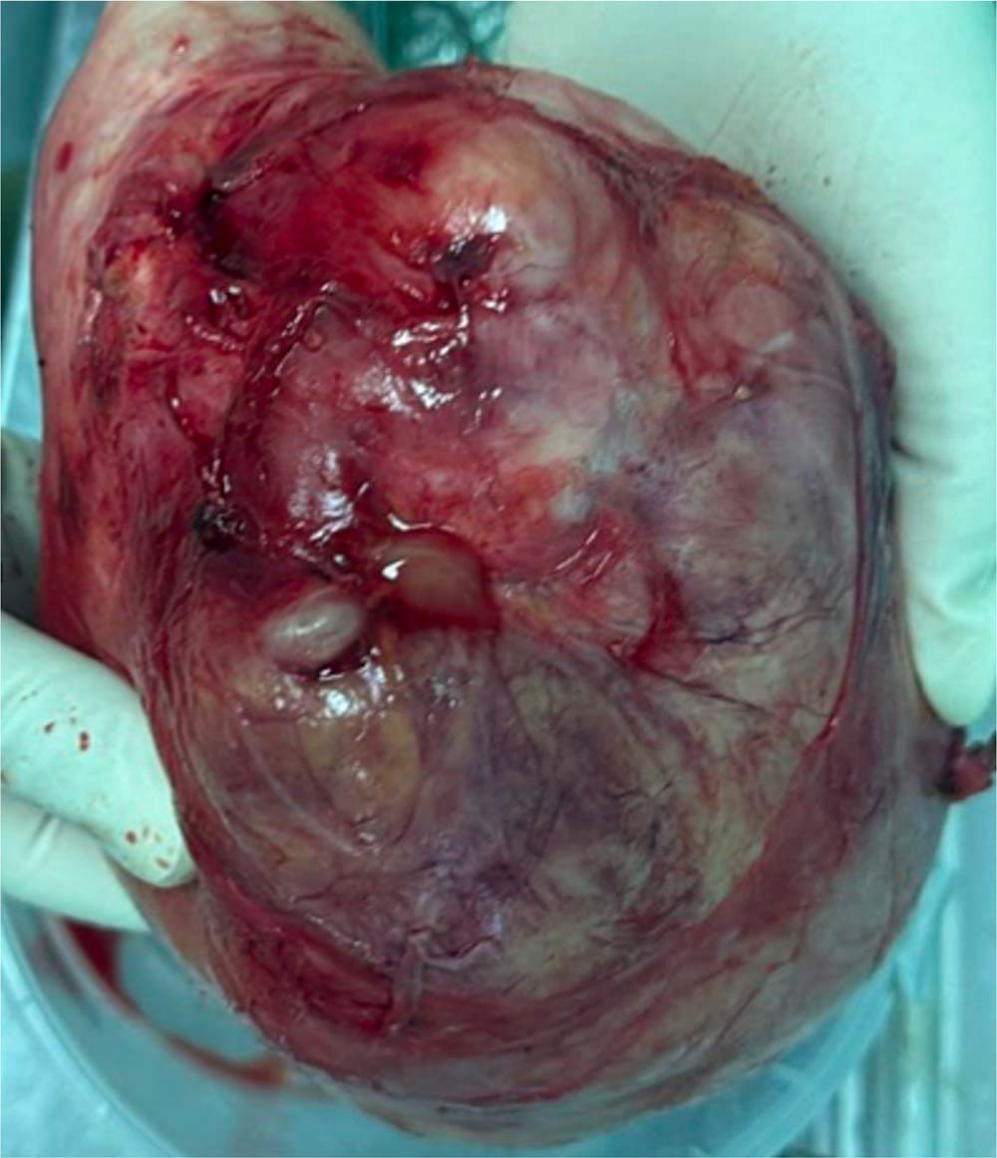
Surgical specimen of the pelvic mass.

Complete surgical excision of the mass was performed through an abdominal approach, along with a total hysterectomy and adnexectomy for definitive treatment and histopathological assessment (Fig. 3).

Histopathological examination confirmed a mature extragonadal pelvic teratoma with no evidence of malignancy.

## Discussion

Teratomas are common germ cell neoplasms derived from totipotent stem cells capable of differentiating into multiple germ layers. These pluripotent cells are predominantly localized at Hensen’s node, situated anterior to the sacrococcygeal region—explaining the prevalence of this site among extragonadal locations. In adults, however, sacrococcygeal teratomas (SCTs) are exceedingly uncommon, with reported incidence rates ranging from 1 in 40 000 to 1 in 63 000 individuals, and a marked female predominance with a 3:1 female-to-male ratio [4].

Sacrococcygeal teratomas (SCTs) are categorized into four types based on their anatomical location: Type I (46%) is entirely external to the pelvis; Type II (35%) is predominantly external but includes a minor intrapelvic portion; Type III (9%) is primarily located within the pelvis with limited external extension; and Type IV (10%) is entirely confined to the pelvis. Types I and II represent ~80% of SCTs and are most frequently observed in neonates, often allowing for early detection and surgical intervention. In contrast, adult SCTs are more commonly classified as Type III or IV [5].

These tumors are predominantly benign and cystic, with malignant transformation occurring in only 1%–2% of cases. The likelihood of malignancy increases with patient age [6]. Histopathologically, sacrococcygeal teratomas are classified into three categories: mature teratomas composed entirely of well-differentiated adult-type tissues; immature teratomas containing embryonic elements; and malignant teratomas, which include malignant germ cell components alongside mature and/or immature tissues [2].

Adult patients with sacrococcygeal teratomas are often asymptomatic at initial presentation, with the lesion incidentally detected during routine digital rectal examination. When symptoms do occur, they may include rectal or colonic obstruction, presenting as constipation or alternating bowel habits, as well as bladder outlet obstruction leading to urinary tract infections or bladder distention—as observed in our case. A minority of patients may develop neurological deficits, such as lower limb weakness or paresthesias, typically in advanced stages when there is malignant infiltration. In contrast to neonatal SCTs, which are externally visible in up to 90% of cases, adult forms are usually confined to the intrapelvic region [7].

Due to the complex anatomical location of sacrococcygeal teratomas, obtaining a biopsy is technically challenging. In addition, the heterogeneous tissue composition of these tumors makes histological evaluation difficult. When malignancy is suspected, preoperative percutaneous or transrectal biopsy is generally discouraged because of the potential risk of tumor seeding, recurrence, or metastasis [5].

Ultrasound is a widely accessible, cost-effective, and safe imaging modality, making it a valuable tool for routine screening. On ultrasound, sacrococcygeal teratomas typically appear as heterogeneous masses containing both solid and cystic components. Areas of increased echogenicity within the lesion may suggest the presence of fat or calcifications, particularly when accompanied by posterior acoustic shadowing [8]. Malignant sacrococcygeal teratomas typically present as solid or mixed solid–cystic masses. Color Doppler ultrasound frequently reveals increased vascular flow within solid malignant lesions. However, increased blood flow may also be observed in rapidly growing, yet benign, SCTs. Arteriovenous fistulas are uncommon. Varying degrees of vascular signals can be detected in solid components of both benign and malignant tumors [5].

CT and MRI are highly effective in detecting lesions within the presacral space. These imaging techniques allow precise evaluation of the relationship between the mass and adjacent structures such as the rectum and sacrococcygeal region, providing valuable guidance for surgical planning. MRI offers superior specificity and accuracy in delineating the soft tissue extent of sacrococcygeal teratomas [5]. Sacrococcygeal teratomas comprise varying amounts of soft tissue, fat, calcifications, and fluid. CT imaging effectively identifies fat and calcifications, with typical attenuation values ranging from −80 to −20 Hounsfield units (HU) for adipose tissue and 80–300 HU for calcium, facilitating clear differentiation. Although CT is highly sensitive for detecting calcifications, which are observed in over 50% of malignant cases, similar findings can also be seen in benign teratomas, limiting their specificity as a marker of malignancy. Consequently, the presence of calcification alone is not a reliable indicator for distinguishing between benign and malignant tumors [3].

Mature teratomas typically appear as predominantly cystic lesions with substantial fatty components and minimal solid parenchyma. On MRI, these tumors often present as round or irregularly lobulated cystic masses exhibiting homogeneous high signal intensity on both T1- and T2-weighted images. Fat-suppressed MRI sequences demonstrate significant signal attenuation within the lesion, indicative of intratumoral fat content—an imaging hallmark of teratomas [5].

Fluorine-18 fluorodeoxyglucose positron emission tomography imaging may be utilized to assess for potential malignant transformation; however, its diagnostic value can be limited, as demonstrated in this case. Moreover, serum tumor markers typically have limited diagnostic value in the assessment of sacrococcygeal teratomas [9].

The differential diagnosis of sacrococcygeal masses in adults depends on the lesion’s characteristics. Unilocular cystic lesions in the presacrococcygeal region may correspond to anterior meningoceles, rectal or anal duplication cysts, or cysts arising from anal glands. In the appropriate clinical setting, fluid collections such as seromas or urinomas should also be considered. In the presence of a multiloculated cystic lesion within the presacral space, a tailgut cyst—also referred to as a retrorectal cystic hamartoma—should be strongly considered in the differential diagnosis. More complex or denser lesions may suggest chronic retrorectal abscesses, pilonidal or dermoid cysts, or primary soft tissue or osseous tumors. Imaging findings of bone destruction or local invasion raise suspicion for malignancy, including sarcomas, chordomas, or metastatic disease. Additionally, sacral osteomyelitis can mimic these entities by producing bony erosion with an associated soft tissue component. Regardless of lesion type, sacrococcygeal teratoma should remain within the differential diagnosis [10].

The primary treatment for sacrococcygeal teratoma is complete surgical excision, which is typically associated with a favorable prognosis. In cases of large tumors, preoperative embolization may be employed to minimize intraoperative blood loss. Surgical access can be achieved via a transabdominal, transperineal (often in the jack-knife position), or combined approach, depending on tumor extent and location. If the coccyx is involved, coccygectomy is often indicated, as the bone may harbor residual pluripotent cells, posing a risk for recurrence.

Benign sacrococcygeal teratomas generally have an excellent prognosis when completely resected, although a small lifetime risk of local recurrence remains. In contrast, malignant forms are associated with a less favorable outcome, often necessitating adjuvant therapy—such as chemotherapy or radiotherapy—tailored to the tumor’s histopathological features [3].

## Conclusion

Sacrococcygeal teratomas develop from pluripotent embryonic germ cells situated in the fetal coccygeal area. Although most commonly diagnosed in infants and children—and sometimes identified prenatally—cases in adults are exceedingly rare and usually represent lesions present since birth that were detected later in life [2]. Imaging modalities, particularly MRI, play a critical role in accurately characterizing these tumors and guiding surgical planning. Although uncommon, sacrococcygeal teratoma should be included in the differential diagnosis of adult female patients presenting with perineal or pelvic masses [6].
